# Continuous retinoic acid induces the differentiation of mature regulatory monocytes but fails to induce regulatory dendritic cells

**DOI:** 10.1186/1471-2172-15-8

**Published:** 2014-02-18

**Authors:** Zachary C VanGundy, Mireia Guerau-de-Arellano, Julie D Baker, Heather R Strange, Susan Olivo-Marston, Dillon C Muth, Tracey L Papenfuss

**Affiliations:** 1 Department of Veterinary Biosciences, The Ohio State University, College of Veterinary Medicine, 370 Veterinary Medical Academic Building, 1900 Coffey Road, Columbus, OH 43210, USA; 2 Division of Medical Laboratory Science, School of Health and Rehabilitation Sciences, School of Health and Rehabilitation Science, The Ohio State University, Columbus, OH 43210, USA; 3 Division of Epidemiology, College of Public Health, The Ohio State University, Columbus, OH 43210, USA

**Keywords:** Regulatory myeloid cells, Dendritic cells, Retinoic acid, Monocyte

## Abstract

**Background:**

Myeloid cells (MC) have potent immunoregulatory abilities that can be therapeutically useful to treat inflammatory disease. However, the factors which promote regulatory myeloid cell differentiation remain poorly understood. We have previously shown that estriol (E3) induces mature regulatory dendritic cells *in vivo*. To determine whether additional steroid hormones could induce mature regulatory myeloid cells, we investigated the effects of retinoic acid (RA) on MCs. Retinoic acid is a steroid hormone important in regulating mucosal immunity in the gut and promoting myeloid differentiation. We hypothesized that the presence of RA during differentiation would promote the formation of mature regulatory myeloid cells (MC_regs_).

**Methods:**

To determine RA’s ability to induce regulatory myeloid cells_,_ we differentiated bone marrow progenitor cells with granulocytic-macrophage colony-stimulating factor (GM-CSF) under the influence of RA. We found that day 7 MCs differentiated in the presence of RA had an increase in the percent positive and relative expression levels of both maturation (CD80, CD86, and MHCII) and inhibitory (PD-L1 and PD-L2) markers compared to control cells. Functionally, these day 7 RA MCs expressed increased intracellular IL-10, induced regulatory T cells *in vitro* compared to controls and suppressed the proliferation of responder immune cells even after inflammatory challenge with LPS.

**Conclusion:**

RA induced mature regulatory myeloid cells that were suppressive and had a CD11b^+^ CD11c^-^Ly6C ^low/intermediate^ monocyte phenotype. Surprisingly, RA CD11c^+^ dendritic cells were not suppressive and could contribute to enhanced proliferation. These results suggest that continuous RA has unique effects on different myeloid populations during monopoeisis and dendropoiesis and promotes a population of regulatory monocytes.

## Background

Myeloid cells (MCs) are a diverse population of cells that form during hematopoiesis and play a critical role in host defense. Comprised of granulocytes, mononuclear phagocytes and their precursors, MCs are innate immune cells that have an important role in promoting inflammation and the induction of adaptive immune responses. Inflammatory MCs are induced and increased in numbers following exposure to exogenous (e.g. pathogens) or endogenous “danger” (e.g. post-necrotic release of high-mobility group box 1 or HMGB1) signals b [1]. These and other environmental factors present within peripheral tissue and bone marrow impact granulopoiesis, monopoiesis and dendropoiesis to influence the ultimate fate of inflammatory granulocytes, monocytes/macrophages and DCs, respectively. While inflammatory MCs have been well characterized, within the last several years the potent regulatory abilities of these cells has increasingly been recognized. Such regulatory MCs (MC_regs_) are a diverse population of cells with the ability to control inflammation and, thus, are a promising target to treat a wide array of inflammatory diseases. To date, however, factors involved in the differentiation of MC_reg_ populations remain poorly understood.

MC_reg_ subsets are particularly diverse, both in terminology and in function. Regulatory, tolerogenic, type II or steady-state are terms applied to regulatory populations of DCs, macrophages, monocytes, and their precursors [1-3]. By and large, the regulatory abilities of macrophages, and more recently, DCs have been most thoroughly studied. First described over 30 years ago, alternatively activated macrophages are able to promote wound healing and resolve inflammation [4,5]. Over the last 10 years the regulatory abilities of DCs and their therapeutic potential have been the focus of many studies [2,6]. Monocytes are circulating myeloid cells that give rise to tissue macrophages and DCs. Monocytes have been recognized as a contributor to the inflammatory responses, and are now known to contribute to immune regulation [7]. MC_regs_ can regulate immune responses through the production of soluble regulatory factors (e.g. IL-10, TGF-beta, indoleamine 2,3 deoxygenase (IDO), arginase, nitric oxide (NO), etc.), expression of inhibitory or regulatory cell surface molecules (e.g. PD-L1, PD-L2) and induction other regulatory cells (e.g. regulatory T cells; T_regs_) or enhance regulatory feed-back loops [8,9]. At present, MC_regs_ are identified based on combination of phenotype and function, with no equivalent to T_reg_ FoxP3 marker being as yet identified [10-13]. Through cell-cell interactions and the production of soluble immunoregulatory molecules, MC_regs_ have very potent and diverse means of inducing immune regulation. However, much remains to be characterized about factors controlling MC_reg_ induction and how different MC_reg_ subsets regulate immune responses. Given that MC_reg_ therapy has the potential to diminish disease in the 100+ millions of individuals impacted by immune-mediated, chronic inflammatory and autoimmune diseases worldwide, it is critical to determine the factors which govern the induction and function of these cells [14-16]. The therapeutic potential of MC_regs,_ has been described in several experimental models of inflammatory and autoimmune disease. Specifically, MC_regs,_ including MDSC, conventional DCs, lung-resident tissue macrophages, monocytes, and plasmacytoid DCs have all been shown to impact disease course in animal models of diabetes [17], colitis [18], allergic asthma [19], experimental autoimmune disease [20], and rheumatoid arthritis [21] respectively.

For many MC_regs_, an arrest in immature and/or altered functionality contributes to their regulatory abilities [22,23]. Glucocorticoids, vitamin D and IL-10 are the most common means to induce these immature MC_regs._ These altered MC_regs_ cells have decreased expression levels of maturation/activation markers CD80, CD86 and MHC class II [2,24-28]. Additionally, these immature MC_regs_ can have reduced inflammatory cytokine expression [29,30], overall blunted function, induce T_regs_ and suppress the action of other immune cells. However, a primary concern with using immature MC_regs_ for therapy is that they may mature into inflammatory MCs under inflammatory disease conditions. Such inflammatory MCs could then actually exacerbate the very inflammatory disease they were used to treat [2,22,23,31]. Thus, mature (and stable) MC_regs_ may avoid such concerns but, to date only a handful of studies have significantly explored the induction of such mature MC_regs_[18,22,32]. Typically, mature MC_regs_ have been induced by combining traditional immature MC_reg_ induction protocols with the addition of inflammatory stimuli such as LPS or TNF-alpha [33,34]. Our laboratory has focused on identifying non-inflammatory systems to induce mature MC_regs_ and we have previously found that estriol (E3), a steroid hormone of pregnancy, produce mature activated DC_regs_[35]. These E3 DC_regs_ maintained their regulatory abilities within an inflammatory environment and protected mice against the inflammatory autoimmune disease, experimental autoimmune encephalomyelitis (EAE) [35]. Although E3 shows promise, the fact that there are limitations on using estrogens broadly in the human patient population necessitated investigating alternative means of inducing mature stable MC_reg_ populations.

All-trans retinoic acid (RA) is a steroid hormone metabolite of vitamin A that plays both an important role during embryonic development and has recently been identified as the key metabolite regulating immune responses at mucosal sites [36-38]. RA is a logical candidate for inducing mature MC_regs_ given its defined role in both mucosal immunoregulation and its ability to promote myeloid cell differentiation and maturation. Within the gut, RA influences the balance between T_regs_ and Th17 cells, B cell isotype switching, antibody production and mucosal homing of numerous immune cells [6,37,39-43]. Mucosal myeloid cells are largely responsible for producing local RA which acts in a paracrine and autocrine manner to regulate mucosal immune responses [6,37]. Although mucosal DCs produce much of the RA required for immune regulation at mucosal sites, much less is known about RA’s direct impact on MC populations at both mucosal and non-mucosal sites [9,19,39,40].

RA regulates myeloid cell survival and promotes the differentiation of immature myeloid cells into mature populations of DCs, macrophages and granulocytes [18,44-46]. Additionally, RA appears to be required for the production of mature phagocytes in the bone marrow through its effects on MHC class II and co-stimulatory molecule expression [47]. Therapeutically, RA has long been used to treat myeloid leukemia given that it promotes myeloid cell differentiation and maturation [48,49]. More recently, it has been used to promote the differentiation of immature myeloid cells (i.e. myeloid derived suppressor cells; MDSCs) in cancer patients to diminish immunosuppressive MDSC effects [36,44,50-53]. Given RA’s important roles in both mucosal immunoregulation and myeloid cell differentiation we hypothesized that RA would induce mature MC_regs_.

Using an *in vitro* model to induce differentiation of MC populations (i.e. DCs, macrophages and monocytes), we evaluated the ability of RA to generate mature MC_regs_[42,54]. We demonstrated that bone marrow cells differentiated with GM-CSF for 7 days in the presence of RA had an activated regulatory phenotype (i.e. increased CD80, CD86, MHC class II, PD-L1 and PD-L2), produced increased IL-10, increased the induction of T_reg_ and suppressed the proliferation of responder immune cells. We found that the suppressive population was a small but potent CD11b^+^ CD11c^-^ Ly6C^low/intermediate^ population whose phenotype is consistent with a regulatory monocyte. Surprisingly the CD11c^+^ DCs were not suppressive. Taken together these results demonstrate a differential effect of RA during monopoiesis and dendropoiesis which results in the induction of regulatory monocytes but not regulatory DCs.

## Results

### Differentiation with retinoic acid induced mature activated regulatory myeloid cells

**Figure 1:**
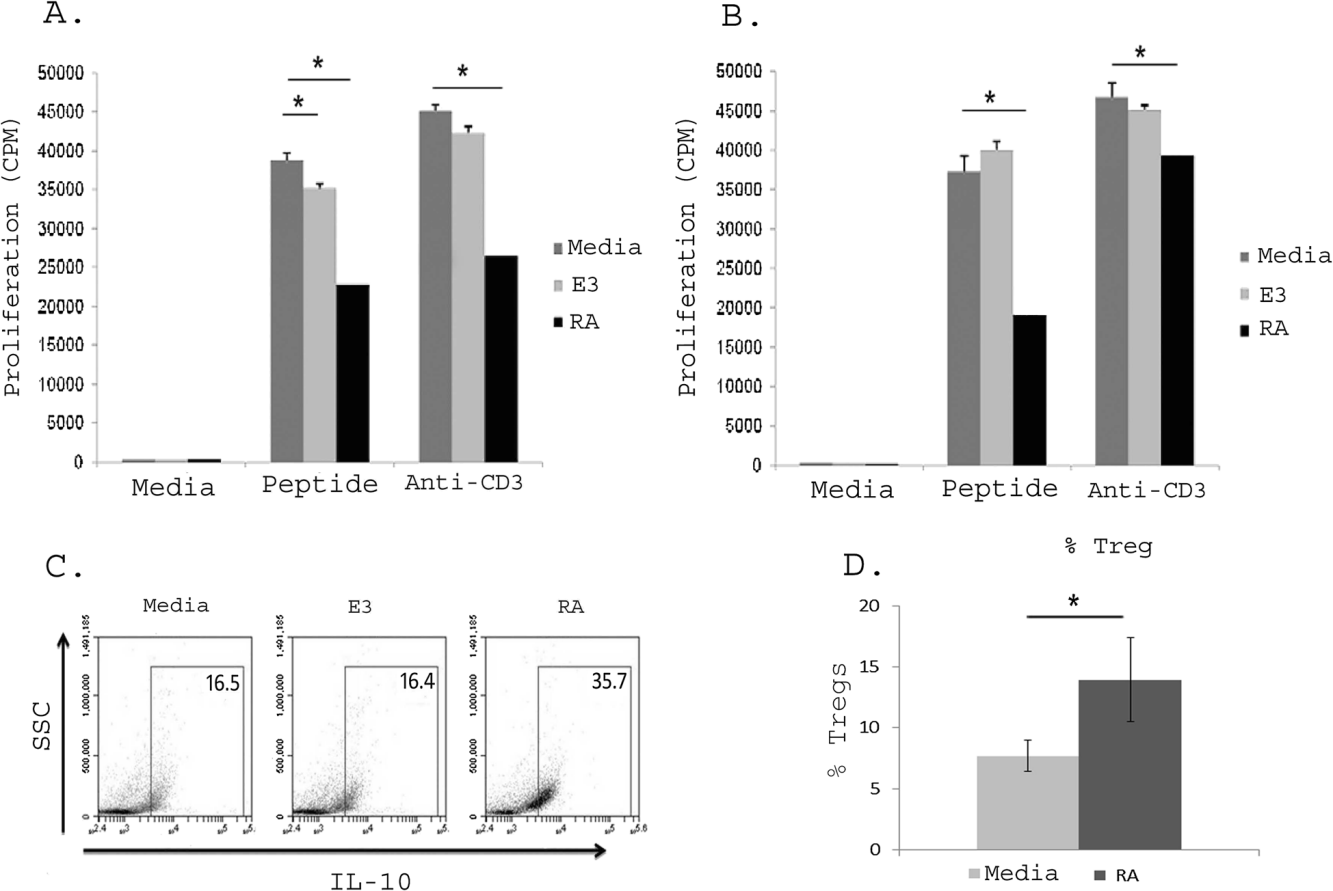
**RA treatment of bone marrow myeloid cells produces a regulatory myeloid cell population.** Bone marrow cells were differentiated in the presence of GM-CSF with or without 100 nM of either estriol or retinoic acid over 6–7 days of differentiation to generate MCs, E3 MC_regs_ or RA MC_regs_. A portion of these cells were also challenged with LPS in the last 24 hours of differentiation. BM-MCs **(A)** and LPS-stimulated BM-MCs **(B)** were co-cultured with responder immune cells containing T cell receptor transgenic CD4^+^ T cells specific for peptide for 96 hours with media, antigen or anti-CD3 stimulation and then pulsed with H^3^ thymidine in the final 18 hours of culture. In MCs, E3 MC_regs_ and RA MC_regs_, **(C)** the relative percentage of IL-10^+^ cells was determined and **(D)** the ability of these cells (after a 5 day co-culture) to induce FoxP3^+^ cells from naïve FoxP3-EGFP reporter immune cells was determined by flow cytometry. Data are representative of at least three separate experiments * = p < 0.05.

Given that RA is a regulator of mucosal immunity and influences myelopoiesis, we hypothesized that RA would induce a population of mature MC_regs_. Day 6–7 BM cells differentiated with GM-CSF in the presence of RA were able to suppress the proliferation of responder immune cells and this suppression was markedly greater than either control or E3 treated cells (Figure 1A). The ability of RA differentiated cells to suppress proliferation was apparent regardless of whether responder immune cells were stimulated with either peptide or anti-CD3. Interestingly, cells treated with E3 suppressed proliferation after stimulation with peptide but not anti-CD3 (Figure 1A). We next determined whether the RA differentiated cells remained regulatory when exposed to the inflammatory stimulus LPS. Figure 1B shows that RA differentiated cells maintained their ability to suppress proliferation even after exposure to LPS challenge and that this was present following stimulation of co-cultures with either peptide or anti-CD3. This effect was entirely lost in E3 treated cells. These results suggest that RA differentiated cells are more potent and stable than E3 differentiated cells and that RA differentiated cells maintain their regulatory ability following exposure to an inflammatory stimulus.

Given that increased IL-10 is seen in E3 DC_regs_[35] and other MC_reg_ populations [50,55] we next evaluated whether RA induced an increase number of IL-10^+^ cells. Figure 1C shows that RA differentiated cells had an increased percentage of IL-10-producing cells compared to either media or E3 control cells. We next evaluated whether RA differentiated cells could increase T_reg_ numbers. We found that RA differentiated cells were able to induce a significant increased percentage of FoxP3^+^ cells following a 5 day culture with naïve immune cells (Figure 1D). Cells differentiated *in vitro* in the presence of E3 failed to significantly increase either IL-10^+^ cells or induce T_reg_ cells (Figures 1C, D). These results show that RA differentiated cells suppressed the proliferative abilities of responder immune cells and induced FoxP3^+^ (T_reg_) cells.

**Figure 2:**
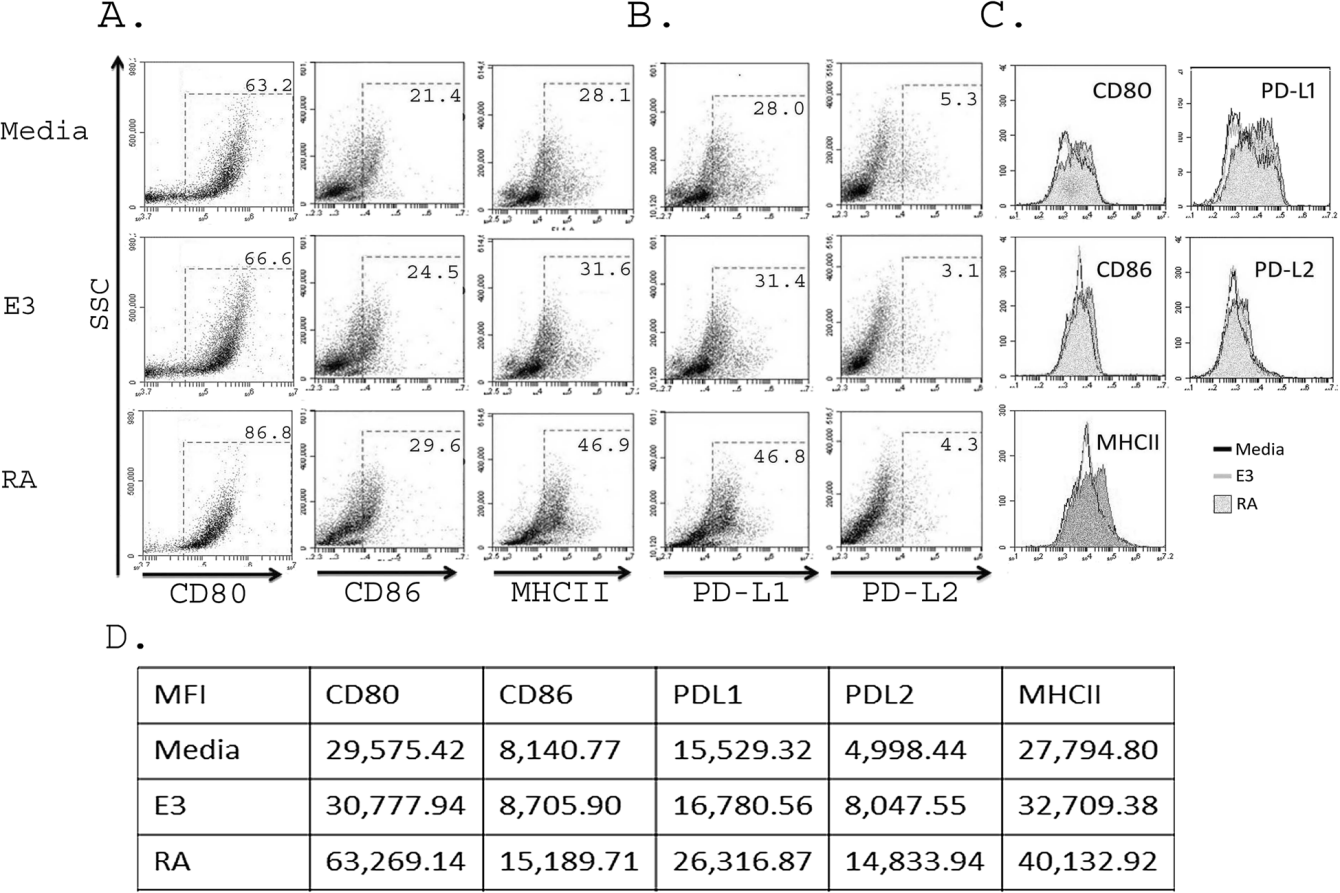
**RA treatment of bone marrow myeloid cells produces mature myeloid cells.** Bone marrow cells were differentiated in the presence of GM-CSF with or without 100 nM of either estriol or retinoic acid over 6–7 days of differentiation. Cells were routinely stained for flow cytometry using antibodies against CD11c-, CD11b^+^, CD80, CD86, MHC class II, PD-L1 and PD-L2, run of a BD Accuri C6 flow cytometer and phenotypic profiles evaluated using the Cflow plus software program. Cells were evaluated for the presence of maturation (activation) markers **(A)** CD80, CD86 MHCII, and the inhibitory co-stimulatory markers **(B)** PD-L1and PD-L2. **(C)** Mean fluorescence intensity (MFI) was determined and relative expression shown by overlays of cell treatment groups and MFI values **(D)**. Data are representative of at least three separate experiments.

To determine whether these RA differentiated cells were mature, we evaluated the cell surface expression of maturation markers CD80, CD86 and MHC class II and inhibitory markers PD-L1 and PD-L2. RA differentiated cells demonstrated an increased percentage of CD80^+^, CD86^+^ and MHC class II^+^ (Figure 2A), indicating that an increased proportion of the cells were mature and/or activated in comparison to E3 or control cells. Additionally, there were increases in the mean fluorescence intensity (MFI) of CD80, CD86 and MHC class II in RA differentiated cells as depicted in Figures 2C and D, indicating that the relative expression levels on a per cell basis were increased in RA differentiated cells. Although E3 differentiated cells had mildly increased expression levels of CD80, CD86 and MHC class II, RA differentiated cells had consistently higher levels than either E3 differentiated or control cells. To confirm that RA differentiated cells demonstrated an “activated regulatory” phenotype as previously described for E3, we evaluated the expression of inhibitory co-stimulatory molecules PD-L1 and PD-L2 [35]. RA increased the percentage of PD-L1^+^ cells (but not PD-L2^+^) (Figure 2B) and the MFI of both PD-L1 and PD-L2 (Figure 2C, D) compared to E3 or media controls. These results demonstrate that during differentiation RA induces a population of mature activated MC_regs_ that suppress the proliferation of responder immune cells even in the face of inflammatory challenge. Additionally, our data shows that although both RA and E3 may induce MC_regs_ which suppress proliferation (Figure 1A) RA MC_regs_ appear to have superior regulatory abilities compared to E3 MC_regs_.

### CD11b^+^ but not CD11c^+^ cells were the suppressive population

**Figure 3:**
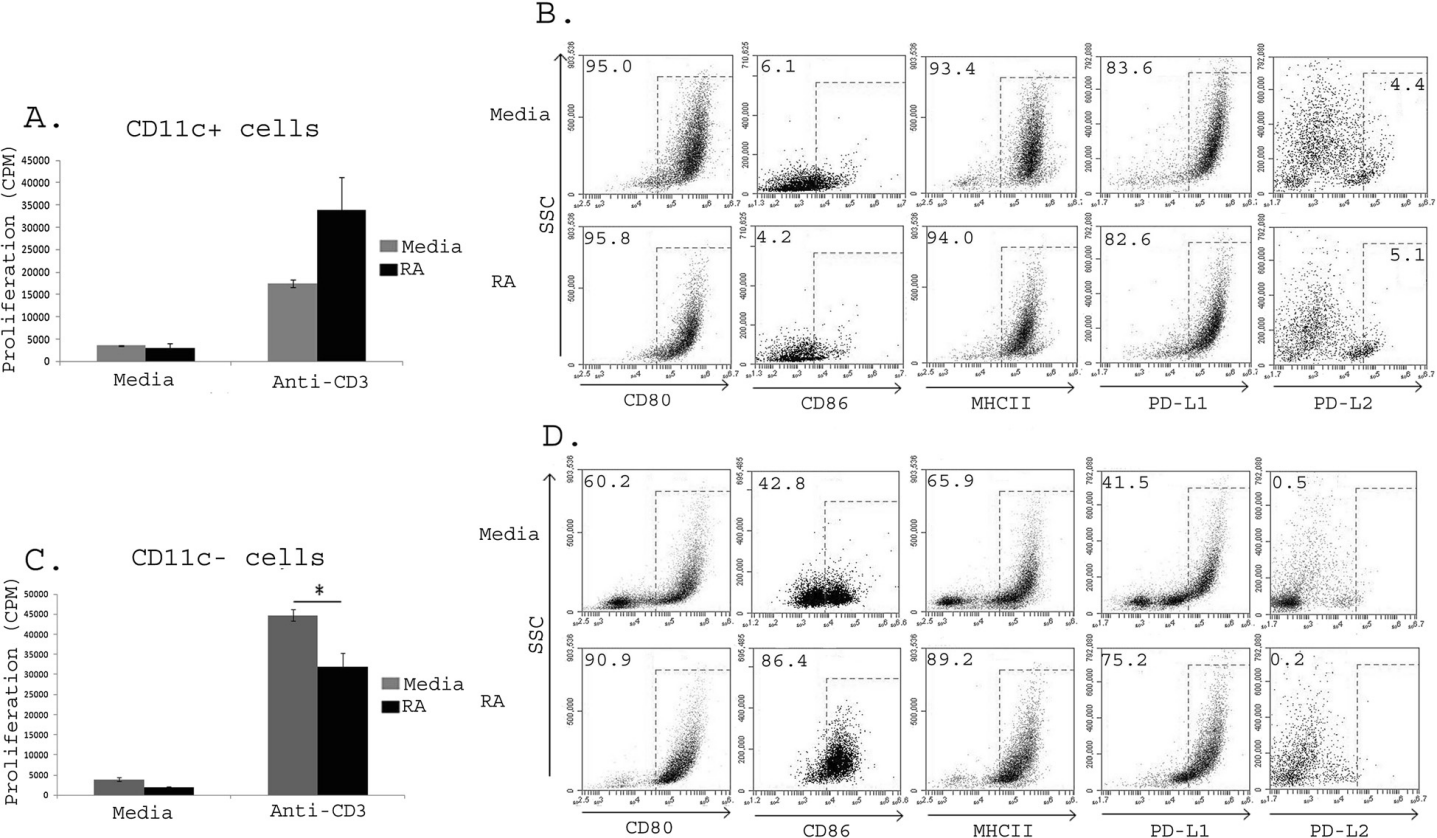
**RA mediated suppression of T cell proliferation is not mediated by CD11c**^**+ **^**BM-MCs.** BM-MCs were magnetically separated with CD11c^+^ beads. Purity of CD11c^+^ and CD11c^-^ cells was confirmed and cells analyzed on the BD Accuri C6 Flow cytometer, Purified CD11c^+^**(A)** and CD11c^-^**(C)** were co-cultured with responder immune cells for 96 hours with media or anti-CD3 stimulation and then pulsed with H^3^ thymidine in the final 18 hours of culture; and the relative percentages of CD11cs^+^**(B)** and CD11c^-^**(D)** cells expressing maturation markers CD80, CD86 MHCII, PD-L1and PD-L2 were determined. Data are representative of three separate experiments* = p < 0.05.

The *in vitro* differentiation of bone marrow cells with GM-CSF is a commonly used protocol to produce large numbers (>80%) of highly enriched CD11c^+^ DCs [38,56] that, as a population, are considered immature DCs. However, our data demonstrated that while approximately 80-90% of the cells were CD11c^+^, the remaining 10-20% were CD11c^-^ but still CD11b^+^ (Additional file 1: Figure S1A). To determine whether the MC_regs_ induced by RA were DCs, we purified CD11c^+^ cells from day 7 differentiated cells and cultured them with responder immune cells. Although RA induction of mucosal “DC_regs_” have been described [9,36,57], we found that RA-treated CD11c^+^ cells were not the suppressive cell population (Figure 3A). In all experiments, RA-treated CD11c^+^ cells failed to suppress proliferation and had variable to no effect on proliferation with some experiments actually demonstrating enhanced proliferation (data not shown). Phenotypic evaluation of these CD11c^+^ cells showed no difference in percentage (Figure 3B) or expression levels of CD80, CD86, MHC class II, PD-L1 and PD-L2 compared to media controls. To determine the source of the suppressive MC_regs_, we evaluated the CD11c^-^ population and found that the RA CD11c^-^ cells suppressed proliferation of responder cells (Figure 3C). These CD11c^-^ cells had a marked (>30%) increase in the percentage of CD80^+^, CD86^+^, MHC class II^+^ and PD-L1^+^ cells (with no differences in PD-L2^+^ cells) (Figure 3D) when differentiated with RA, consistent with an activated regulatory phenotype in these cells described previously [35]. In contrast, levels of CD80, MHC class II and PD-L1 did not change, remaining consistently high (>80%) in RA versus control MCs. (Figure 3B). These data suggest that RA present during GM-CSF differentiation increased an activated regulatory phenotype in the CD11c^-^ (non-DC) populations.

**Figure 4:**
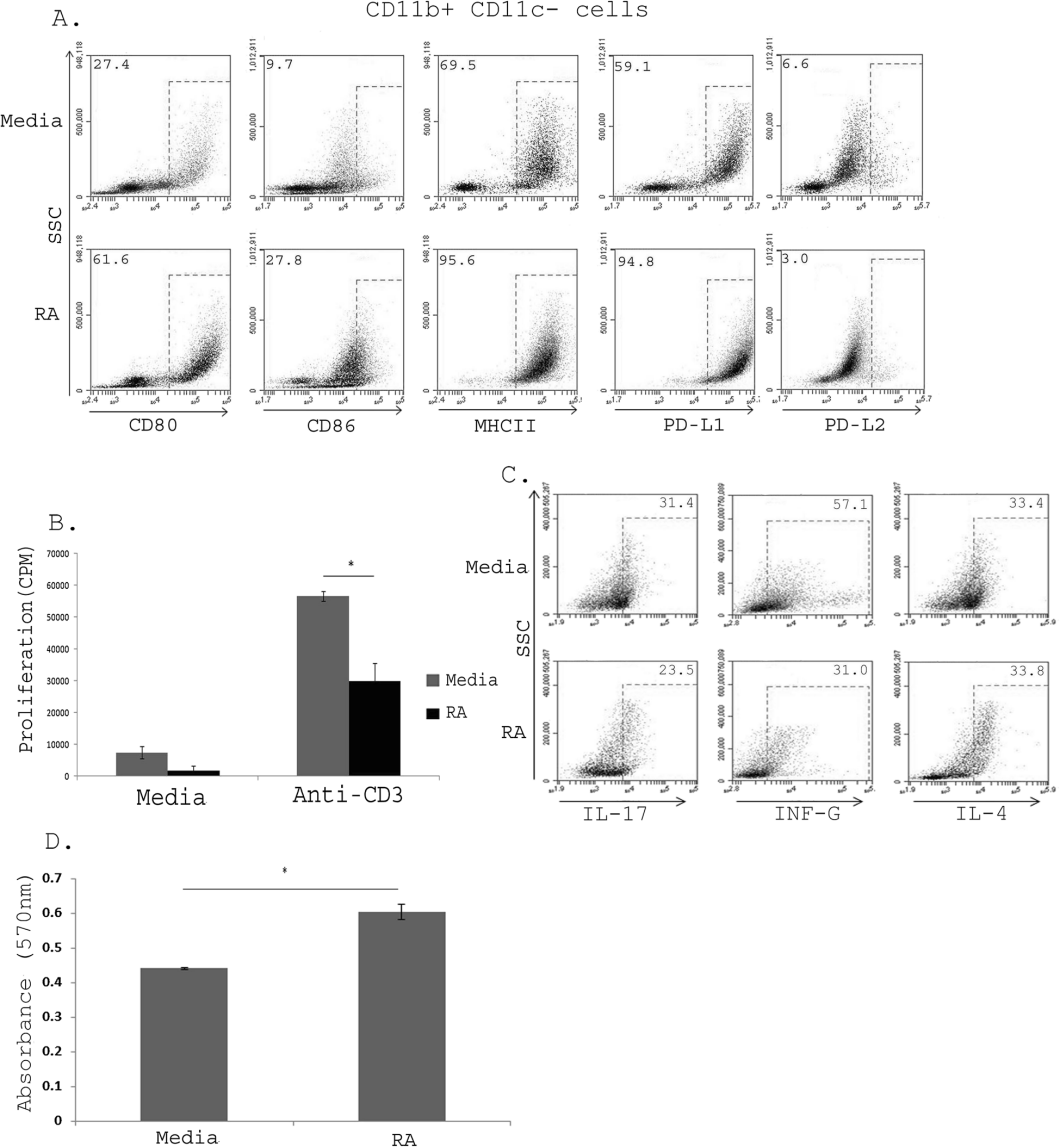
**RA generated CD11b**^**+ **^**BM-MCs suppress T cell proliferation and express a regulatory phenotype.** CD11b^+^ CD11c^-^ were purified from BM-MCs by sequential purification of CD11b^+^ cells from the CD11c- fraction and purity determined to be >95%. Cells were routinely stained for cell surface markers and flow cytometry performed using the BD Accuri C6 Flow cytometer. CD11b^+^ CD11c cells were **(A)** assessed for expression of maturation markers CD80, CD86, MHCII, PD-L1 and PD-L2 **(B)** co-cultured with responder immune cells for 96 hours with media or anti-CD3 stimulation and then pulsed with H^3^ thymidine in the final 18 hours of culture, **(C)** intracellular cytokine staining (IL-17, INF-γ, IL-4) of CD4^+^ cells performed and **(D)** co-cultured with activated CTLs and a T lymphoma cell line with cell viability assessed by the MTT assay. Data is representative of three separate experiments. * = p < 0.05 **(A-C)** Data shown is a representation of 3 experiments **(D)**.

Both differentiated and precursor populations within the bone marrow are predominantly but not completely CD11b^+^ (>90%) (Additional file 1: Figure S1A). To definitively isolate the effects of CD11b^+^ CD11c^-^ cells, we serially purified CD11b^+^ cells from the CD11c^-^ fraction and evaluated their phenotype and function. As expected, the increases in the percentage of CD80^+^, CD86^+^, MHC class II^+^ and PD-L1^+^ cells seen in Figure 3D was also seen in the CD11b^+^ CD11c^-^ serially purified population (Figure 4A). We then went on to evaluate the ability of these cells to influence CD4 and CD8 responses. We found that the CD11b^+^ CD11c^-^ population was able to suppress the proliferation of responder immune cells (Figure 4B) and could modify the cytokine profile of T cells. The proliferating CD4^+^ responder immune cells cultured with RA CD11b^+^ CD11c^-^ cells were also shown to have reduced expression of IL-17 IFN-gamma (Figure 4C) and IL-10 (Additional file 2: Figure S2) but no change in IL-4 production as determined by intracellular cytokine staining (Figure 4C). Intracellular IL-10 and FoxP3^+^ cells were also increased as expected (Additional file 2: Figure S2A and S2B, respectively). We also evaluated the ability of RA CD11b^+^ CD11c^-^ cells to influence CD8^+^ T cell responses. Figure 4D demonstrates reduced cytotoxicity in CD8^+^ T cells cultured with RA CD11b^+^ CD11c^-^ cells. Taken together, these results suggest that RA induced an activated regulatory population of CD11b^+^ CD11c^-^ cells that were able to suppress both CD4^+^ and CD8^+^ adaptive immune responses.

### CD11b^+^ CD11c-Ly6C^low/intermediate^ were the primary population responsible for suppression

**Figure 5:**
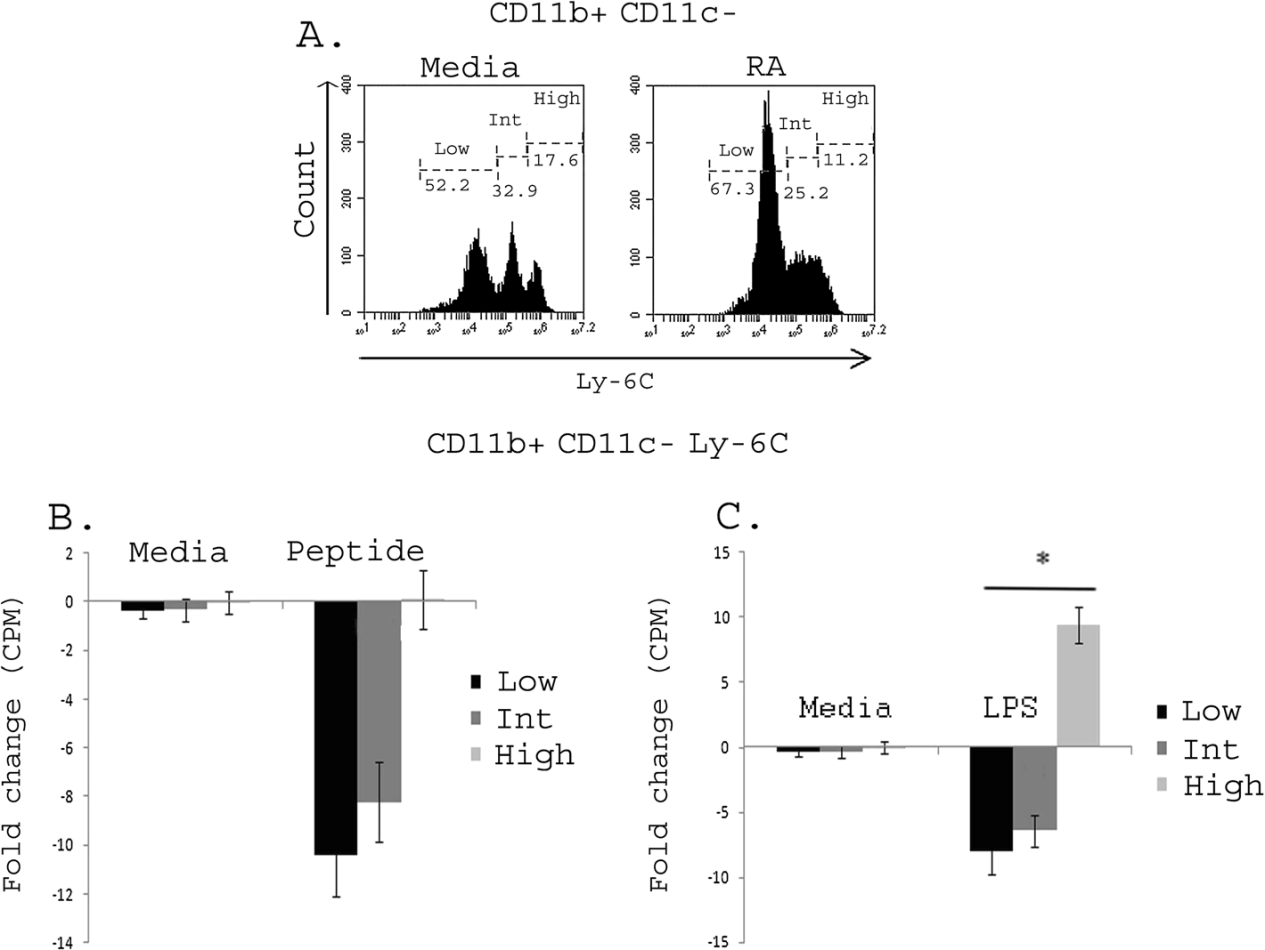
**RA CD11b**^**+ **^**CD11c-Ly6C**^**low/intermediate **^**are the suppressive population.** BM cells were differentiated in the presence of GM-CSF with or without 100 nM RA for 7 days, stained with fluorescently-labeled antibodies against CD11c, CD11b^+^, and Ly-6C to determine relative expression of Ly6C on CD11b^+^ CD11c cells **(A)**. Cells were then sorted based on relative Ly6C expression and purified populations co-cultured with antigen-specific T cell receptor transgenic T cells for 96 hours with media, peptide or LPS and fold change in proliferation shown for peptide-stimulated **(B)** or LPS-stimulated **(C)** co-cultures. Data are representative of at least three separate experiments. * = p < 0.05.

**Figure 6:**
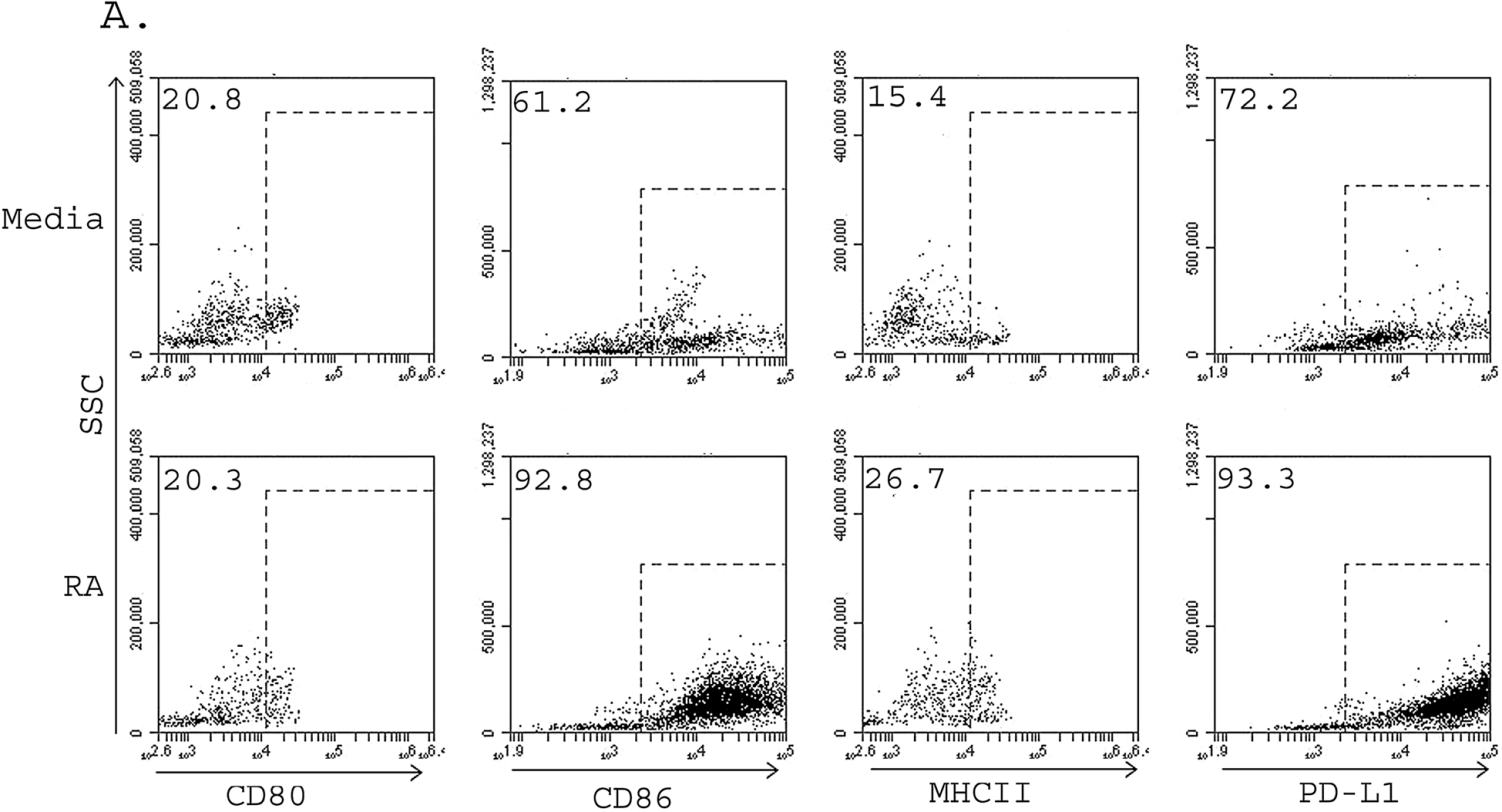
**CD11b**^**+ **^**CD11c**^**- **^**Ly-6C**^**low **^**cells have an activated regulatory phenotype.** BM-MCs were differentiated with or without 100 nM RA for 7 days. Myeloid populations were separated by Flow sorting on the FACS Aria III by antibodies CD11c and Ly-6C^low/int^ and extracellular markers (CD80, CD86, MHCII, and PD-L1) were assessed on the Accuri C6 flow cytometer. Data are representative of three separate experiments.

Although used primarily to induce large numbers of DCs, differentiation with GM-CSF can potentially promote the differentiation of a mixture of granulocytes, monocytes, macrophages and DCs [42,54,56,58-60] In our GM-CSF cultures, we found that Ly-6G^+^ granulocytes were no longer present in CD11b^+^ cells at day 7 of differentiation (Additional file 1: Figure S1B), indicating that granulocytes were not responsible for the suppression seen [61,62]. To determine whether monocytes were present and may be responsible for the suppressive effects, we evaluated day 7 non-adherent cells sorted based on their relative expression of the monocyte marker Ly-6C. Ly-6C expression levels have been shown to correlate with cellular function and maturation level where Ly-6C^high^ monocytes are inflammatory and Ly-6C^low^ monocytes are steady-state or regulatory [7,63]. Figure 5A shows that the presence of RA during differentiation increased the percentage of cells expressing low to intermediate levels of Ly6C. To determine whether the increase in these cells was responsible for the suppression seen in the CD11b^+^ CD11c^-^ population, we sorted cells based on Ly-6C^low^, Ly-6C^intermediate^, and Ly-6C^high^ expression patterns. Figure 5B demonstrates that both Ly-6C^low^ and Ly-6C^intermediate^ cells were able to suppress up to a 6-fold decrease in proliferation of responder cells following antigenic stimulation while Ly-6C^high^ cells failed to influence peptide-specific proliferation. Similarly, Ly-6C^low^, Ly-6C^intermediate^ cells maintained their ability to suppress proliferation (Figure 5C) even when co-cultures were stimulated with LPS. In contrast, Ly-6C^high^ actually significantly increased the proliferation of responder immune cells (Figure 5C) following stimulation with LPS. These results demonstrate that RA Ly-6C^low^ and Ly-6C^intermediate^ cells are the suppressive population and are able to maintain suppressive abilities even in the presence of inflammatory (LPS) challenge. Phenotypically, RA Ly-6C^low^ cells showed the most marked increase in the percentage of PD-L1^+^, as well as, CD86^+^ and MHC class II^+^ cells, with over 90% of the Ly-6C^low^ cells expressing PD-L1 (Figure 6). A similar but less dramatic phenotype was seen in the Ly-6C^intermediate^ cells (data not shown). Taken together, these data show that RA induces a small but potent population of CD11b^+^ CD11c^-^ Ly-6C^low/intermediate^ MC_regs_ consistent with an activated regulatory monocyte phenotype that are able to suppress immune cell proliferation.

## Discussion

The principal objective of this study was to determine whether RA, a steroid hormone known to play important roles in regulating both mucosal immune responses and differentiation of myeloid cells could generate an activated (or mature) MC_reg_ population. We demonstrate that RA influences myelopoiesis to a regulatory MC_reg_ (monocyte) with the phenotype of CD11b^+^ CD11c-Ly-6C^low/intermediate^ but fails to induce DC_regs_. These cells can influence both CD4^+^ and CD8^+^ responses and promote FoxP3^+^ (T_reg_) cell induction. Our data suggest that RA has distinctly different effects on monopoiesis and dendropoiesis to promote the generation of regulatory monocytes.

MC_regs_ are a diverse population of cells and much attention has focused on the *in vitro* generation and clinical application of MC_regs_. While the *in vitro* generation of such MC_reg_ populations has great therapeutic potential, much remains to be learned regarding the factors which contribute to MC_reg_ induction. The majority of *in vitro* generated MC_regs_ are arrested in an immature or hypo-functional state. An emerging concern is that these immature MC_regs_ populations may mature to become inflammatory DCs or macrophages and, thus, contribute to inflammatory disease pathology [2,22,23,29-31,64]. A more recent approach is to induce mature MC_regs_ which would be stable and maintain regulatory potential in an inflammatory environment [22,32,65,66]. Anderson and colleagues have demonstrated that human DC_regs_ (generated with dexamethasone, vitamin D and LPS) maintain tolerogenic activity and actually induce significantly higher levels of IL-10 production by resultant T cells [33]. However, the relative stability and ability of MC_regs_ (such as DC_regs_) to maintain regulatory abilities during inflammation may still be in question. For example, a study by Voigtlander *et al.* suggests that DC_regs_ induced by TNF-alpha do not maintain their regulatory abilities upon a secondary stimulation with TNF-alpha *in vivo*[34]. Obviously, this is of considerable concern given that TNF-alpha is present in a large array of inflammatory conditions where such DC_regs_ (or other MC_reg_ populations) may be applied therapeutically. Much work remains to determine critical factors important in generating mature MC_regs_ for anti-inflammatory therapies but we have focused on non-inflammatory pathways to induce mature MC_regs_.

We have shown that mature MC_regs_ can be generated with the use of steroid hormones alone [35]. Our previous work has shown that the sex steroid hormone estriol (E3) induces a mature activated MC_reg_ population of CD11c^+^ DC_regs_ that protects against inflammatory challenge *in vitro* and in an *in vivo* disease model [35]. In the present study, we have extended our research of pathways involved in normal homeostatic induction of mature MC_regs_ by investigating the ability of the steroid hormone RA to induce mature MC_regs_ that are resistant to inflammatory challenge. Our results show that RA is more effective than E3 *in vitro* in generating MC_regs_ and that these MC_regs_ are resistant to LPS inflammatory challenge.

RA is known for its ability to promote the differentiation, and maturation, of myeloid cell populations. This ability, along with its known immunoregulatory role at mucosal sites, made it a logical candidate for these studies [44,52]. RA is present in relatively large concentrations within mucosal sites and is largely produced by local antigen presenting cells (APCs) residing within these mucosal sites. Specifically, mucosal CD103^+^ DCs are the primary immunoregulatory myeloid cells within the gut. These DCs have up-regulated *raldh2* gene expression, constitutively produce RA, and produce increased TGF-beta. They also have a significant ability to induce Foxp3^+^ T_regs_, mucosal homing receptors CCR9 and α4β7 expression on lymphocytes and enhance antibody production and Ig isotype switching [6,9,36,57]. These mucosal DCs are the most common MCs investigated regarding RA biology and induced mucosal DCs have been generated from monocytes or splenic DCs with GM-CSF with IL-4 [8,43] or bone marrow precursors with RA [18,41,57,67,68]. Increasingly, the non-mucosal and therapeutic applications of RA (i.e. in cancer) are being investigated [9,19,43,44,53] and this study focused on RA’s ability to induce mature activated MC_regs_ that are able to suppress responder immune cell proliferation [8,35,41,43,57,69].

Given RA’s critical role in DC-mediated immunoregulation within the gut, it was quite surprising that RA CD11c^+^ cells were not suppressive. One possibility is that DC’s differentiated with RA could generate mucosal DCs but wouldn’t generate mature activated DC_regs_ that could suppress proliferation as seen with E3 DC_regs._ While induction of mucosal DCs can be accomplished with RA [18,43], the immunomodulatory abilities of these DCs as described in these studies was not the focus of this study. Alternatively, timing of RA administration may have resulted in the lack of DC_reg_ induction as described by Feng and colleagues [41]. Specifically, their studies showed that the presence of 1 μM RA from day 0 throughout differentiation failed to induce mucosal DCs. Although different dosages and criteria were used to generate and identify DCs as mucosal (versus DC_regs_ in our study), the continuous presence of RA during differentiation may have resulted in the inability to induce DC_regs_ in our study. Similarly, Wada’s group showed that the use of a synthetic RARα and β agonist (AM-80) could differentiate human peripheral blood monocytes into dendritic cells that have a tolerogenic phenotype and function [18]. The use of AM-80 versus ATRA in our study or the differentiation of human monocytes versus murine myeloid progenitors could explain the differences in DC_regs_ versus MC_regs_ in our study.

It could be argued that CD11c^-^ DC precursors existed within the population of CD11b^+^ CD11c^-^ suppressive cells. Given the described effects of RA in promoting differentiation and maturation, in conjunction with our data demonstrating an activated phenotype, we believe this to be unlikely [57,70-72]. Rather, our data on Ly6C expression strongly support that the suppressive cells were regulatory monocytes with an activated regulatory phenotype (increased CD80, CD86, MHC class II and PD-L1) consistent with previous work within our lab. Given that the CD11b^+^ CD11c^-^ population is comprised less than 20% of the entire population, the ability of these cells to suppress both CD4^+^ and CD8^+^ responses is noteworthy. The specific contributions of cell contact-dependent (i.e. PD-L1) versus cell contact-independent (i.e. IL-10, TGF-beta, etc.) mechanisms responsible for the regulatory abilities of these cells was beyond the scope of this study. However, we did see increases in regulatory markers including PD-L1, IL-10 and the percentage of FoxP3^+^ cells with RA MC_regs_.

Monocytes are circulating myeloid cells which give rise to tissue DCs and macrophages, and their regulatory abilities have recently been recognized [7]. Although numerous markers can be present on mouse monocytes (e.g. CD11b, CD115, CCR2, CX3CR1 and Ly-6C), we chose to investigate Ly6C expression levels given that they have been correlated with monocyte function [7,63,71,73]. Specifically, Ly-6C^high^ represents an inflammatory monocytes while, Ly-6C^low/intermediate^ monocytes have been shown to play important roles in patrolling the vasculature and potentially resolving inflammation and tissue repair [7,63,74-76]. Ly6C is also down regulated following differentiation which is consistent with our findings where RA, a molecule known to promote differentiation and maturation, increases the percentage of cells that are Ly-6C^low/intermediate^ (Figure 5A) [3,44]. Our data suggest that Ly-6C levels correlate with suppressive abilities with the lowest Ly-6C expression associated with the most suppressive ability. Given that Ly-6C^high^ monocytes are typically inflammatory monocytes, it is not surprising that proliferation is actually enhanced following LPS stimulation in this cell population (Figure 5C). Taken together, these data showed a progression from Ly-6C^high^ to Ly-6C^low^ associated with increasing regulatory abilities. These results are consistent with the association seen between Ly-6C expression and blood monocyte function described by others [7,63,71,77]. Currently, the mechanisms and pathways by which RA maturation of monocytes imparts them with increased regulatory abilities remain undefined. Whether a specific signal during differentiation drives monocytes to become regulatory in an active process or whether differentiation under homeostatic or regulatory (i.e. RA) conditions in the absence of inflammatory stimuli is a default mechanism for regulatory monocyte induction is unknown. Additionally, whether these RA Ly-6C^low/intermediate^ monocytes have the potential to further differentiate into DC_reg_ or regulatory macrophage populations remains to be determined and is the subject of ongoing studies within the laboratory [7].

## Conclusion

Our findings show that continuous RA exposure during myelopoiesis promotes the induction of MC_regs_. Specifically, RA induced CD11b^+^ CD11c^-^Ly-6C^low/intermediate^ regulatory monocytes which suppressed the proliferation of immune cells but RA failed to induce DC_regs_. Our data suggests that RA has unique effects on different myeloid populations during differentiation that may influence the regulatory abilities of monocytes and DCs. A more thorough understanding of how RA mediates these differential effects has important implications in our understanding of MC_reg_ biology and the potential application of these cells to treat a wide variety of inflammatory diseases.

## Methods

### Mice

C57BL/6 (H-2^b^) mice (4–8 wk old), C57BL/6-Tg (TcraTcrb)425Cbn/J, C57BL/6-Tg(Tcra2D2,Tcrb2D2)1Kuch/J and reporter Foxp3EGFP (B6.Cg-*Foxp3*^*tm2Tch)*^) were purchased from Jackson Laboratories (Bar Harbor, ME, USA) or bred in-house. Mice were housed five per cage and maintained on a 12 hr. light/dark cycle, maintained under specific pathogen-free conditions and were housed and cared for according to the institutional guidelines of the Ohio State University’s Institute for Animal Care and Use Committee.

### Cell lines

EG7 and EL7 (kindly provided by P. Boyaka, Ohio State University) were used to study the MHC class I-restricted response of CTLs in mice. The EG7 cells have been transfected with plasma to synthesize and constitutively secrete OVA 257–264 peptide and should be cultured in 10% RPMI. The EL4 cells are the non-OVA secreting duplicate of the EG7. Both are commonly found at ATCC but were acquired through Dr. Boyaka. The DC2.4 cell line was kindly provided by K. Rock, University of Massachessetts and as a DC antigen-presenting cell.

### BM-MC differentiation and development of regulatory MC differentiation model

Bone marrow (BM) cells were collected from C57Bl/6 mice femurs and tibias. After erythrocyte lysis (AKC or in-house lysis buffer), cells were cultured with RPMI 1640 (Invitrogen) supplemented with 10% FBS, 25 mM HEPES, 2 mM L-glutamine, 50 U/ml penicillin, 50 mg/ml streptomycin, 5 × 10^-5^ M 2-mercaptoethanol and 200U/ml recombinant murine GM-CSF (R&D Systems) ± 100 nM of either estriol (E3) or all-trans retinoic acid (RA) (Sigma-Aldrich) for 6–7 days at a density of 2 × 10^6^ cells/ml. Day 6–7 cells were considered differentiated BM-MCs (media control) and BM-MC_regs_(RA and E3). Cells were challenged with inflammatory stimulus LPS (1 μg/ml, 055:B5, Sigma-Aldrich) during culture as indicated at day 6 or later for BM-DCs.

### Functional immunosuppressive assays: T cell proliferation assay

Myeloid cells (BM-MCs or BM-MC_regs_) were cultured with responder spleen cells from antigen-specific T cell receptor transgenic (TCR Tg; where antigen was either OVA323-339 or MOG35-55) or Foxp3EGFP mice as indicated. To assess T cell proliferation co-cultures were stimulated with anti-CD3 (BD Bioscience), T cell-receptor specific antigen MOG35-55 (Bio Matic) or T cell-receptor specific antigen OVA 323–339 (Anaspec). To assess the effects of myeloid cell activation, co-cultures were stimulated with LPS from Escherichia coli, 055:B5 (Sigma-Aldrich) for 96 hours, pulsed with (H^3^ thymidine) (Perkin Elmer Life Sciences or MP Biomedicals) in the last 18 hours, harvested and counted, data is expressed as counts per million (cpm) ± SEM [35].

### Functional immunosuppressive assay: CD8^+^ cytotoxic assay

To generate CTL, spleen and lymph nodes (LN) were removed from OT-1 mice and co-cultured with OVA (257–264) pulsed DC2.4 cells (kindly provided by Kenneth Rock, University of Massachussets) for 4 days, removed and cultured with mrIL-2 (R&D Systems) for 2 days. OVA-expressing (EG7) and non-transfected control cells (EL4) were seeded at 2 × 10^4^ cells per well and co-cultured with CTLs (1 × 10^5^) and control or RA treated monocytes (2 × 10^4^) for 6–18 hours [73,78]. The MTT assay (Sigma-Aldrich) was used to determine quantity of live cells. Briefly, after incubation, cells were centrifuged (1500 RPM for 5 min) media was decanted and 100 ul of fresh media was added. 10ul of 5 mg/ml thiazolyl blue tetrazolium bromide (MTT) was added to each well for 2 hours at 37°C. After incubation cells were centrifuged (1500 RPM for 7 min) and media was decanted, cells were allowed to dry for 15–30 min before 100 ul of DMSO was added, mixed well and read at 570 nm on a Spectra Max 2. The absorbance levels were calculated by averaging the non-specific and specific absorbance levels of five separate data sets. Media control is compared to RA treated cells.

### *In vitro* T_reg_ induction

Bone marrow (BM) cells were collected from C57Bl/6 mice femurs and tibias. After erythrocyte lysis, BM cells were cultured with supplemented RPMI (as previously described) for 7 days at a density of 2x10^6^ cells/ml +/− RA. Spleens from mice with reporter Foxp3EGFP (B6.Cg-*Foxp3*^*tm2Tch)*^) were harvested, passed through cell strainers (70 μm, BD Falcon), collected by centrifugation (1500 RPM for 7 Min at 4°C) and subjected to erythrocyte lysis. Responder cells and MCs or CD11c^-^ MCs were cultured for 4–6 days and aliquots from cultures assessed for Foxp3 expression by flow cytometry.

### Flow cytometry

*In vivo* and *in vitro* derived DCs and MCs were labeled and evaluated by three-color flow cytometry using combination of the following conjugated directly antibodies (clone): CD11c (HL3), CD11b (M1/70), Gr-1 (RB6-8C5), Ly-6G (1A8), Ly-6C (AL-21), MHC class II (AF6-120.1), CD80 (16-10A1), CD86 (IT 2.2), PD-L1 (MIH5), and PD-L2 (YT25) with appropriate isotype controls. (BD Bioscience, eBiosciences or Miltenyi Biotec). Cells were stained with fluorochrome-labeled antibodies or isotype controls for 20 min in the dark at 4°C, washed twice in FACS buffer and re-suspended in 300 μl FACS buffer for flow cytometry analysis.

Intracellular IL-10, IL-4, IL-17 and INF-γ levels were measured after incubating myeloid cells with 1 μg/ml LPS overnight (IL-10) or Ionomyocin (1 mg/ml) and PMA (25 ng/ml) for 4 hours (IL-4, IL-17, and INF-γ). 2 μM of Monensin (eBioscience) added 2–4 hrs. before harvesting cells. Cells were removed from culture, washed with 2 ml of supplemented RPMI and blocked with 0.5 μg/ml Fc block (anti-CD16/CD32) for 15 minutes. Cells that required extracellular markers were re-suspended in FACS buffer and stained with anti-CD4 (0.2 mg/ml) and incubated in the dark at 4°C for 20 min. Cells were washed with FACS buffer (2x with 1 ml) and then fixed and permeabilized using FIX/PERM solution (BD Bioscience), briefly vortexed and incubated in the dark at 4°C for 20 min. Cells were then washed twice with 1 ml of PERM/WASH buffer (BD Bioscience), re-suspended in PERM/WASH buffer and stained with 0.2 mg/ml anti-IL-10 (BD Bioscience) for 30 min. in the dark at 4°C. All flow samples were processed on an Accuri C6 flow cytometer and results analyzed using the Accuri C6 Flow software (BD Biosciences).

### Myeloid cell purification

Day 6–7 differentiated BM cells were incubated with manufacturer suggested amounts of CD11c/CD11b microbeads (Miltenyi Biotec) for 15 minutes in the dark at 4°C. Cells were washed with running buffer (10% FBS in PBS with 900 mg of NaN_2_ per 1 L of PBS), and centrifuged (1500 RPM, 7Min). Cell separation was performed using either the Auto Macs (Miltenyi Biotec) magnetic separation instrument or the FACS Aria III 12 color, 4 laser cell sorter. The Auto Macs was used according to the manufacturer’s instructions. Cell sorting with the FACS Aria III was performed at the OSU Flow Cytometry Core and isotype control antibodies were included to determine detection levels. CD11b^+^ CD11c^-^ Ly-6C^low^ monocyte populations were serially gated on CD11c^-^ cells, followed by CD11b^+^ with gates set around distinct populations of Ly-6C low, intermediate and high. The purity of the cell populations was ≥95%.

### Statistical analysis

Data are represented as mean +/− SEM or fold change. Statistical significance was determined using a Student’s t-test or 1 way ANOVA with a significance level (p-value) < 0.05 and the Wilcoxen signed-rank test. All analyses were performed using Excel and/or GraphPad Prism software (La Jolla, CA).

## Abbreviations

APC: Antigen presenting cell; GM-CSF: Granulocyte-macrophage colony-stimulating factor; PD-L1: PD-L2, Program death ligand 1 and 2; E3: Estriol; DC: Dendritic cell; tDC/DC_reg_: Tolerogenic/Regulatory Dendritic cell.

## Competing interests

The authors declare that they have no competing of interest.

## Authors’ contributions

ZCV, JDB, DCM, HRS, MG-d-A and TLP performed research and analyzed data. TLP designed the research. SO-M provided statistical data analysis. ZCV and TLP wrote the manuscript. TLP and HRS revised and edited the manuscript. All authors read and approved the final manuscript.

## Supplementary Material

Additional file 1: Figure S1 GM-CSF induced myeloid cells. BM-MCs were differentiated for 6–7 days and characterized phenotypically using the Accuri C6 Flow cytometer to identify the relative percentage of the cell population expressing **(A)** of CD11c^+^ and CD11b^+^ media and RA differentiated cells. Expression of LY-6G was evaluated by the Accuri C6 cytometer of the **(B)** CD11b^+^ cells. Data are representative of at least three separate experiments. Click here for file

Additional file 2: Figure S2 CD11c^-^ IL-10 and T_reg_ cell induction. Bone marrow cells were differentiated in the presence of GM-CSF with or without 100 nM of or retinoic acid over 7 days to generate BM-MCs. Following differentiation MCs were magnetically labeled with CD11c^+^ beads and separated with the AutoMacs. Purity was confirmed by routine staining of positive and negative cells with FITC-conjugated anti-CD11c antibody and cells were run on the Accuri C6 Flow cytometer. **(A)** The relative percentage of IL-10^+^ cells was determined in control MCs and RA MC_regs_. Data are representative of at least three separate experiments. **(B)** Day 7 media CD11b^+^ CD11c^-^ MCs or RA CD11b^+^ CD11c^-^MCs were co-cultured in the presence of Foxp3EGFP reporter cells and expression of Foxp3^+^ cells was evaluated in the lymphocyte population over time in the cultures by flow cytometry. Data shown is a representation of 3 experiments. Click here for file
