# Supplementary figures and images for: Continuous retinoic acid induces the differentiation of mature regulatory monocytes but fails to induce regulatory dendritic cells
Source: BMC Immunol. 2014 Feb 18;15:8. doi: 10.1186/1471-2172-15-8 (PMC4016499; doi:10.1186/1471-2172-15-8)

Supplemental figure 1

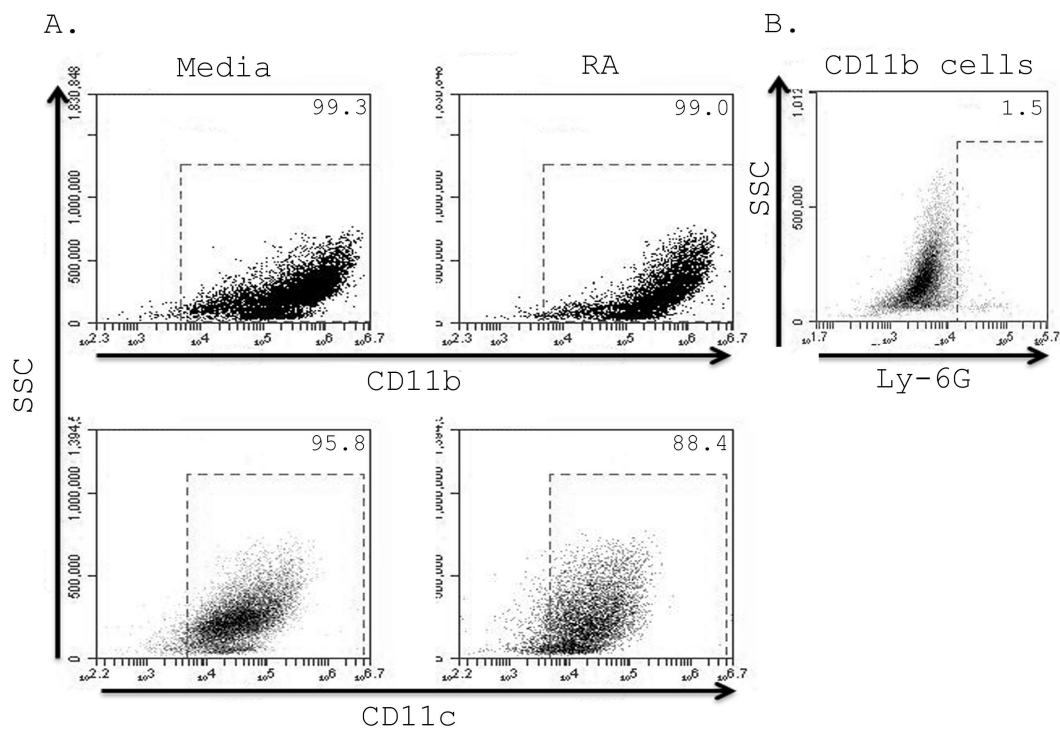

Supplement: Additional file 1: Figure S1 — GM-CSF induced myeloid cells. BM-MCs were differentiated for 6–7 days and characterized phenotypically using the Accuri C6 Flow cytometer to identify the relative percentage of the cell population expressing (A) of CD11c+ and CD11b+ media and RA differentiated cells. Expression of LY-6G was evaluated by the Accuri C6 cytometer of the (B) CD11b+ cells. Data are representative of at least three separate experiments. [file 1471-2172-15-8-S1.pdf]

CD11b+ CD11c-

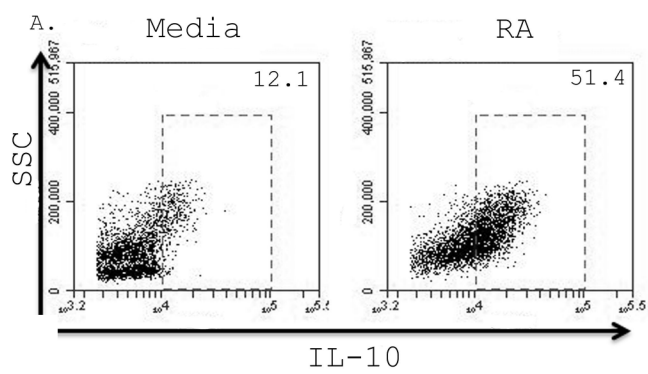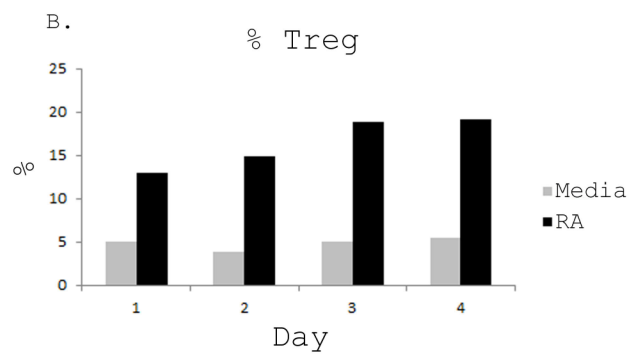

Supplement: Additional file 2: Figure S2 — CD11c- IL-10 and Treg cell induction. Bone marrow cells were differentiated in the presence of GM-CSF with or without 100 nM of or retinoic acid over 7 days to generate BM-MCs. Following differentiation MCs were magnetically labeled with CD11c+ beads and separated with the AutoMacs. Purity was confirmed by routine staining of positive and negative cells with FITC-conjugated anti-CD11c antibody and cells were run on the Accuri C6 Flow cytometer. (A) The relative percentage of IL-10+ cells was determined in control MCs and RA MCregs. Data are representative of at least three separate experiments. (B) Day 7 media CD11b+ CD11c- MCs or RA CD11b+ CD11c-MCs were co-cultured in the presence of Foxp3EGFP reporter cells and expression of Foxp3+ cells was evaluated in the lymphocyte population over time in the cultures by flow cytometry. Data shown is a representation of 3 experiments. [file 1471-2172-15-8-S2.pdf]
